# Imaging Intron Evolution

**DOI:** 10.3390/mps5040053

**Published:** 2022-06-24

**Authors:** Maria Antonietta Panaro, Rosa Calvello, Daniela Valeria Miniero, Vincenzo Mitolo, Antonia Cianciulli

**Affiliations:** Department of Biosciences, Biotechnologies and Biopharmaceutics, University of Bari, Via Orabona, 4, 70126 Bari, Italy; mariaantonietta.panaro@uniba.it (M.A.P.); rosa.calvello@uniba.it (R.C.); danielavaleria.miniero@uniba.it (D.V.M.); mitolo09ottobre@libero.it (V.M.)

**Keywords:** intron evolution, BLAST dot plots, Multiz alignment and conservation tool, SLC25A21, GHR

## Abstract

Intron evolution may be readily imaged through the combined use of the “dot plot” function of the NCBI BLAST, aligning two sequences at a time, and the Vertebrate “Multiz” alignment and conservation tool of the UCSC Genome Browser. With the NCBI BLAST, an ideal alignment of two highly conserved sequences generates a diagonal straight line in the plot from the lower left corner to the upper right corner. Gaps in this line correspond to non-conserved sections. In addition, the dot plot of the alignment of a sequence with the same sequence after the removal of the Transposable Elements (TEs) can be observed along the diagonal gaps that correspond to the sites of TE insertion. The UCSC Genome Browser can graph, along the entire sequence of a single gene, the level of overall conservation in vertebrates. This level can be compared with the conservation level of the gene in one or more selected vertebrate species. As an example, we show the graphic analysis of the intron conservation in two genes: the mitochondrial solute carrier 21 (SLC25A21) and the growth hormone receptor (GHR), whose coding sequences are conserved through vertebrates, while their introns show dramatic changes in nucleotide composition and even length. In the SLC25A21, a few short but significant nucleotide sequences are conserved in zebrafish, Xenopus and humans, and the rate of conservation steadily increases from chicken/human to mouse/human alignments. In the GHR, a less conserved gene, the earlier indication of intron conservation is a small signal in chicken/human alignment. The UCSC tool may simultaneously display the conservation level of a gene in different vertebrates, with reference to the level of overall conservation in Vertebrates. It is shown that, at least in SLC25A21, the sites of higher conservation are not always coincident in chicken and zebrafish nor are the sites of higher vertebrate conservation.

## 1. Introduction

In eukaryotes, the coding DNA sequence of most protein-coding genes is interrupted by the interpolation of non-coding DNA sequences termed introns (“*intragenic regions*”), as opposed to the coding DNA segments termed exons [1,2]. In the cell nucleus, the whole gene is sequentially transcribed as a pre-mRNA, but before it is passed to the cytoplasm as mature mRNA, the introns are removed by a process called RNA splicing, carried out by a large ribonucleoprotein machinery called the Spliceosome [3,4,5]. In evolutionary conserved genes, the sites of splicing are also most often conserved, although alternatively spliced transcripts may be expressed in addition to the canonical transcript [6,7]. Although originally considered “junk” DNA, the introns are now believed to play a role in the control of gene expression [8,9]. In addition, introns are thought to control their own splicing by carrying nucleotide signaling sequences at their ends (5’ss and 3’ss) [10,11], as well as internal splicing enhancer or silencing sequences and sequences that interact with components of the spliceosome [12]. The origin and evolution of introns is still poorly understood. It is debated whether introns are very ancient and are gradually being lost, or whether their relatively recent evolution has caused them to gradually accumulate: the intron-early and intron-late hypotheses, respectively [13,14,15,16,17]. It is likely that introns have played a role in the main steps of animal evolution.

This short graphical essay aims to demonstrate the use of NCBI BLAST dot plots and the UCSC Genome Browser graphics for ready visual demonstrations of evolutionary patterns in introns of homologous genes. Here, we show the evolution—from Euteleostomi to Amphibia, birds and mammals—of two representative genes: the mitochondrial 2-oxodicarboxylate carrier (mitochondrial solute carrier 21; SLC25A21) [18] and the growth hormone receptor (GHR) [19]. Possibly due to the very basic life functions they serve, in the mitochondrial transporter family (SLC25), members of the coding sequences (and protein products) are conserved between fungi and primates; these genes are among the most studied within vertebrates [20,21]. In particular, the SLC25A21 gene was selected as the oldest member of this family in humans. The GHR gene is also “conserved”, but limited to vertebrates.

The sequence evolution in introns has been studied with reference to specific sites or motifs only. For instance, the conservation in homologous 5’ss and 3’ss [7,22] and the occurrence of short k-mer words [23] were investigated in vertebrates. However, from these studies a comprehensive scheme of the intron evolution did not emerge.

Two-sequence BLAST comparisons were made for each gene between the intron sequences of different species, and the results are mainly presented as dot plot diagrams. The genes studied are, in all species, considered heavily burdened with specific transposable elements (TEs) [24] intervening between the genuine original intronic sequences. We thus made additional comparisons between the actual intronic sequence of a gene and the intronic sequence of the same gene after TE clearing, or between TE-cleared introns of two different species. This procedure points out the sites of TES as blanks in the alignment.

The UCSC Genome graphics was used to display, for SLC25A21 or GHR, along their entire sequence, the average level of conservation calculated from up to 100 multialigned vertebrate species. In addition, the conservation of the relevant gene in selected vertebrate species was graphed in parallel and this allowed both comparisons among species and comparisons between individual species and the vertebrate average.

The extensive information that may be gained from analyses of the type outlined here might help in drafting a scheme of the evolutionary flow of the information carried through introns. The primary information concerning protein structure is conveyed by exons, which maintain a relative structural stability through the negative selection of modified key nucleotides. As a result, key proteins are conserved through very long evolutionary periods, as in SLC25 genes. Introns possibly carry supplemental information, regulating the gene expression, for example. The results of the present investigation show that the structural conservation in introns is much poorer than in exons, but the mutation rate during evolution may be very different depending on the gene concerned. Furthermore, the insertions of species-specific TEs contribute to the structural divergence between species. In conclusion, the information carried by introns (if any) is likely to fade out quite rapidly, albeit with some differences between genes.

The graphical approaches discussed in this paper have the advantage of readily providing quick comparative overviews of the intron structure of two or more homologous genes, as well as the possibility of zooming in on specific segments. Under the guidance of the visual information gained, further quantitative analyses may focus on regions of specific interest, for instance, pertaining to the conservation of nucleotide composition or motifs, the similarity of secondary structures or the presence of TEs common to different species.

## 2. Material and Methods

The NCBI bank of homologous genes (https://www.ncbi.nlm.nih.gov/homologene, accessed on 1 January 2020) was used to select the homologous SLC25A21 and GHR genes of human (*Homo sapiens*), mouse (*Mus musculus*), chicken (*Gallus gallus*), zebrafish (*Danio rerio*), and Xenopus (*Xenopus tropicalis*). Genomic and mRNA sequences of the genes were retrieved from the NCBI GenBank (https://www.ncbi.nlm.nih.gov, accessed on 1 January 2020) (Table 1 and Table 2). The genomic sequences (from start codon to stop codon) were processed by deleting the coding sequences, thus leaving the introns only (fourth column of Table 3). Some analyses were performed after clearing the intron sequences of all transposable elements (TEs), using the CENSOR software (https://www.girinst.org/censor/, accessed on 1 January 2020) [25] (Table 3, last column). Nucleotide alignments were usually performed with BLAST (Basic Local Alignment Search Tool; https://blast.ncbi.nlm.nih.gov/Blast.cgi, accessed on 1 January 2020), setting the Expect threshold at 0.0005. BLAST finds regions of local similarity between sequences and calculates the statistical significance of matches [26]. The dot plot is a graph on the XY plane, each dot having the positions of the aligned segment in one gene and in the other gene as coordinates. When comparing homologous genes, the series of dots will align along a somewhat regular straight line stretching from the lower left corner to the upper right corner. The gaps in the line correspond to non-homologous segments.

The UCSC Genome Browser was accessed at https://genome.ucsc.edu on 1 January 2020, and the function “Comparative Genomics-Conservation” was used. This method shows multiple alignments of 100 vertebrate species and measurements of evolutionary conservation using two methods (phastCons and phyloP).

Further instructions for both BLAST and UCSC Genome Browser plots are available at the indicated sites.

BLAST dot plots and UCSC graphics photos are unretouched in order to show the actual outputs of the analysis. However, when the matching “dots” were too small to be viewed on the original print (e.g., human/zebrafish alignments, accessed on 1 January 2020), the plots were redrawn with suitably sized dots at the sites of alignment.

The percentages of nucleotide conservation between species were calculated as follows: From the list of the individual aligned sections (with their length) provided by BLAST, we computed the total number of the aligning nucleotides and divided it by the total number of intron nucleotides.

## 3. Results

### 3.1. The TEs

**Figure 1:**
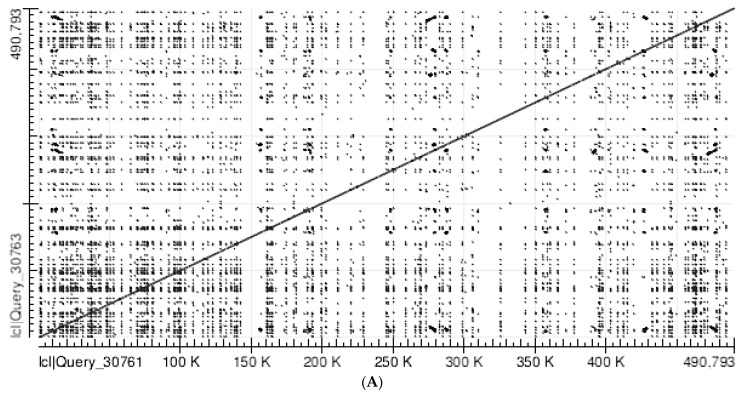

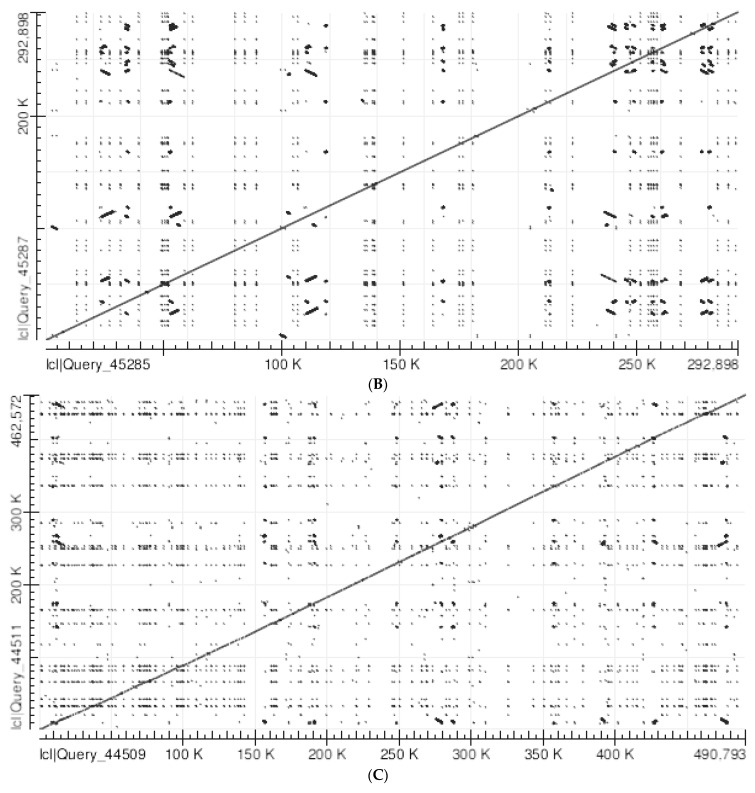

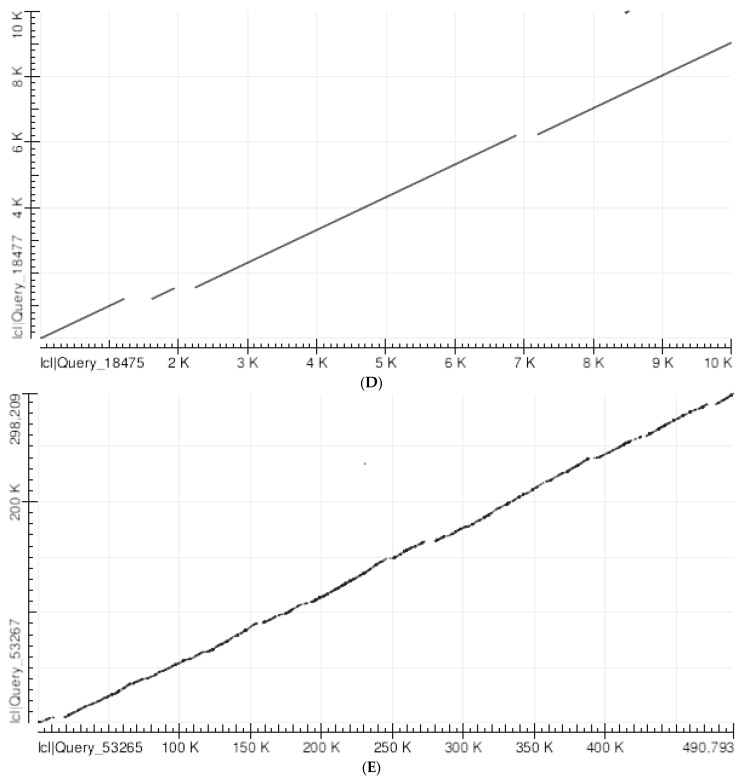
(**A**) Abscissa: Human SLC25A21 (490,793 nucleotides; the sequence from start to stop codons after removal of exons). Ordinate: the same sequence as in the Abscissa. Number of matches: 7704. Note the perfect alignment along the diagonal from the lower left corner to the upper right corner and the large number of matches outside the diagonal. (**B**) Abscissa: Human GHR (292,898 nucleotides; the sequence from start to stop codons after removal of exons). Ordinate: the same sequence as in the Abscissa. Number of matches: 1840. Note the perfect alignment along the diagonal from the lower left corner to the upper right corner and the large number of matches outside the diagonal. (**C**) Abscissa: Human SLC25A21 (490,793 nucleotides). Ordinate: Human SLC25A21 after removal of all the 130 SINE *Alu*s (462,572 nucleotides). Number of matches: 3220 (compared with **A**). Note the seemingly perfect alignment along the diagonal from the lower left corner to the upper right corner. (**D**) A partial, magnified, view of panel (**C**), showing a 10 K per 10 K area from the lower left corner, magnified by 50×. The gaps correspond to the removed SINE *Alu*s. (**E**) Abscissa: Human SLC25A21 (490,793 nucleotides). Ordinate: Human SLC25A21 after removal of all TEs (298,209 nucleotides). These additional TEs belong to different categories, and some of them are much larger than the SINE *Alu*s. Number of matches: 505. Note that the gaps (due to TEs) along the diagonal from the lower left corner to the upper right corner are now more visible at low magnification.

The alignment of an intron sequence with itself, apart from the expected exact diagonal alignment, usually shows a large number of matches outside the diagonal (Figure 1A,B).

In the next experiment, we removed the SINE *Alu*s from the SLC25A21 intron sequence which are the most represented TEs in human genes, but average 280 nucleotides only in length. Their removal generates a dramatic decrease in the matches outside the diagonal but also reveals gaps along the diagonal. Due to the short length of the *Alu*s, the gaps in the diagonal are not apparent at a low magnification (Figure 1C), but may be clearly demonstrated at a higher magnification (Figure 1D). When all the other non-*Alu* TEs were also removed (a total of 192,584 nucleotides, i.e., about 39% of the introns) the small gaps along the diagonal could be appreciated, even at a low magnification. It was also seen that all matches outside the diagonal disappeared, showing that the latter were all due to coupling between TEs (Figure 1E). The same experiment with GHR yielded similar results (not shown).

### 3.2. Human/Mouse Alignments

**Figure 2:**
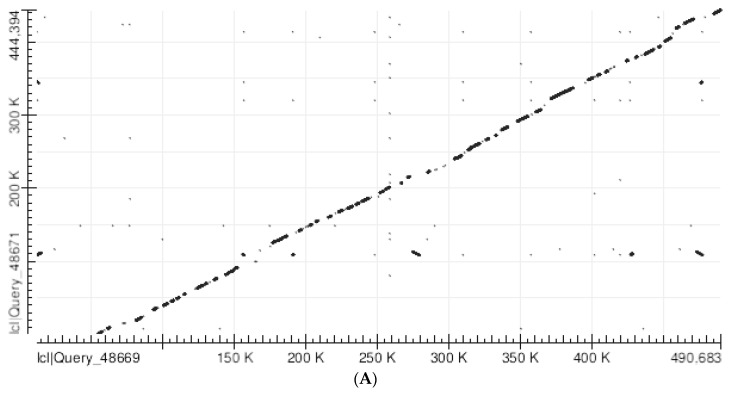

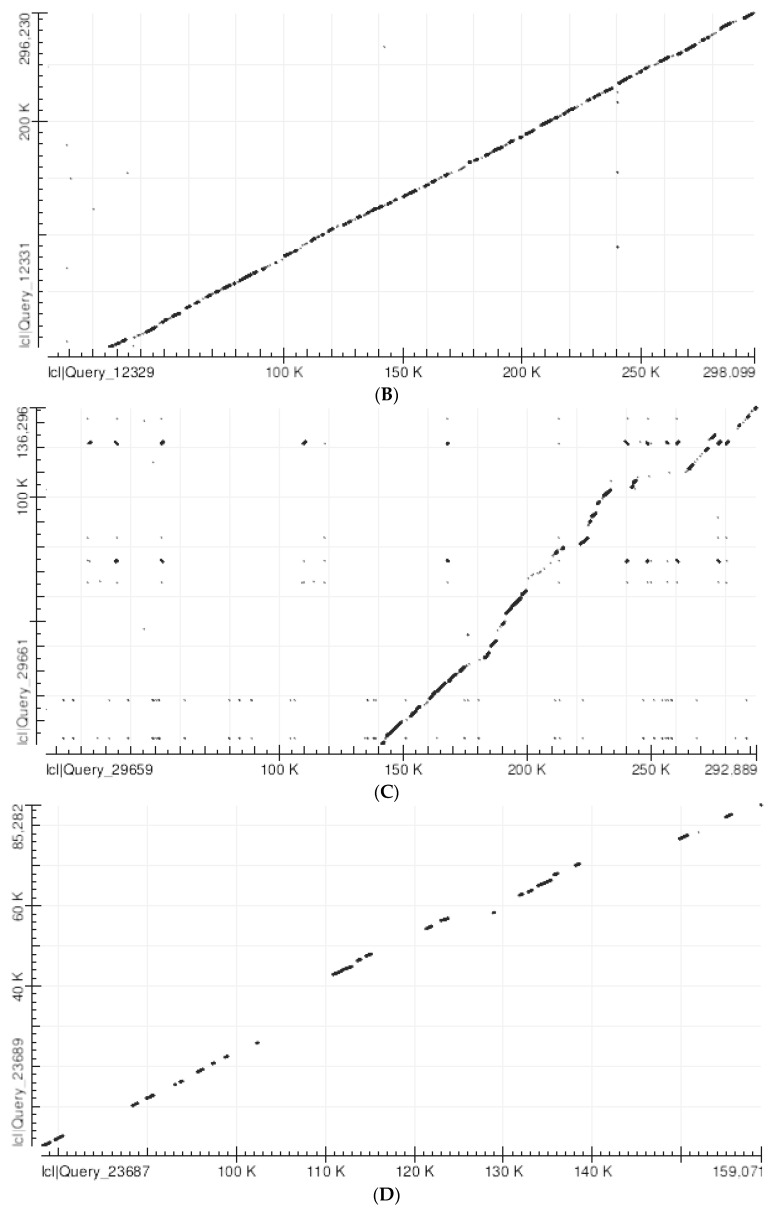
(**A**) Abscissa: Human SLC25A21 (490,793 nucleotides). Ordinate: Mouse slc25a21 (444,514 nucleotides). Number of matches: 1660. Note the significant alignment of most matches along the diagonal from the lower left corner to the upper right corner. The matches which are outside the diagonal mostly correspond to human and mouse TEs similar in structure. (**B**) Abscissa: Human SLC25A21 after the removal of all TEs. (298,209 nucleotides). Ordinate: Mouse slc25a21 after the removal of all TEs. (296,350 nucleotides). Number of matches: 266.The plot demonstrates a good overall alignment between the two sequences, while the gaps (which are usually short) correspond to poorly conserved sections. Note also the absence of significant matches outside the diagonal. (**C**) Abscissa: Human GHR (292,898 nucleotides). Ordinate: Mouse GHR (136,305 nucleotides). Number of matches: 280. Note the significant alignment of most matches along the diagonal from human GHR nucleotide 140,000 to the upper right corner, indicating that human nucleotides 1 to 140,000 have no matches with the mouse sequence, while the rest of the human sequence and the whole mouse sequence are homologous. The matches that are outside the diagonal mostly correspond to TEs that are similar in human and mouse. (**D**) Abscissa: Human GHR (homologous section only) after the removal of all TEs (85,058 nucleotides; from 78,000 to163,058). Ordinate: Mouse GHR after the removal of all TEs (89,777 nucleotides). Number of matches: 27. The plot demonstrates a general overall alignment between the two sequences, but the gaps are numerous and wide, corresponding to poorly conserved sections.

The BLAST dot plot alignment of the human SLC25A21 with the homologous mouse gene shows that the large majority of matches lie along a slightly irregular diagonal line. The additional sparse matches are likely to correspond to TEs, which are at least partly similar in human and mouse, such as LINE 1 transposons (Figure 2A); indeed, when all TEs are cancelled in both genes before the alignment, the sparse matches are practically absent (and the alignment diagonal is more regular) (Figure 2B). The gaps (usually short) in the alignment diagonal correspond to poorly conserved sections.

The BLAST dot plot alignment of the human GHR with the homologous mouse gene shows that approximately 1 to 140,000 human nucleotides have no match with the mouse sequence and are likely to be an additional insertion peculiar in human. On the contrary, the rest of the human sequence significantly aligns with the mouse sequence, although the alignment line is considerably distorted and exhibits relatively large gaps (Figure 2C). Following the removal of all TEs in both sequences, the diagonal alignment becomes more regular, but with gaps that are much larger than in the SLC25A21 alignments (Figure 2D; compare with Figure 1E).

### 3.3. Human/Chicken Alignments

**Figure 3:**
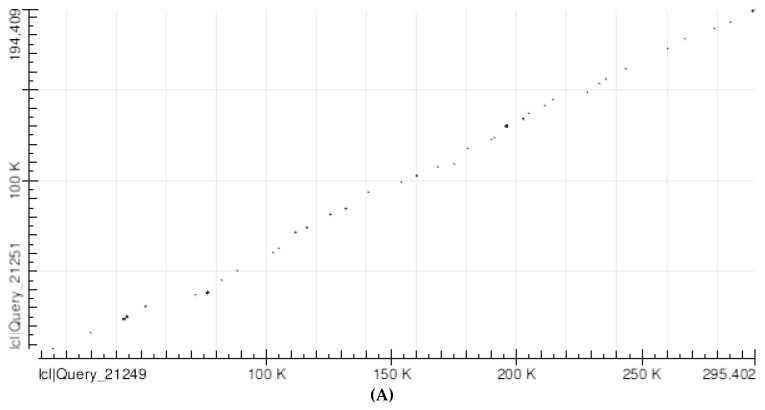

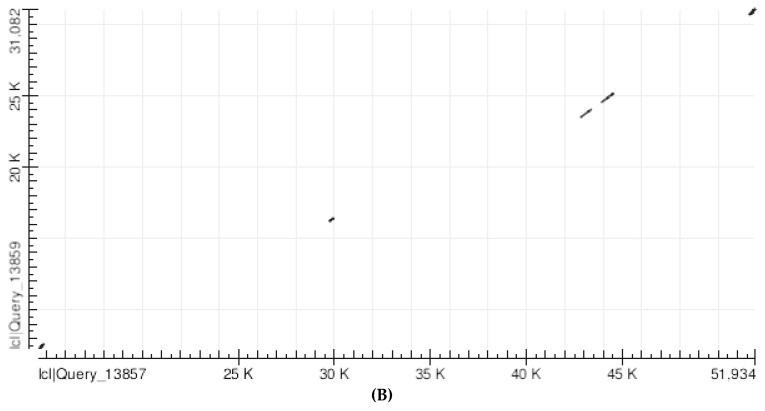
(**A**) Abscissa: Human SLC25A21 after the removal of all TEs (298,209 nucleotides). Ordinate: Chicken slc25a21 after the removal of all the TEs (200,341 nucleotides). Number of matches: 32. Short but likely significant matching segments align almost exactly along the diagonal from the lower left corner to the upper right corner. Gaps between matching segments are relatively wide and correspond to segments that do not share significant homologies. Chicken nucleotides aligning with human: 10,717 over 230,188, i.e., 4.66%. (**B**) A detail (magnification 5.7 times) of the lower left corner of (**A**) (52,000 by 32,000 nt).

The BLAST dot plot alignment of the human SLC25A21 with the homologous chicken gene yielded virtually identical results, whether using the complete sequences or after TEs deletion, since practically no TE is shared between the two species. Figure 3A shows that the vast majority of matches, i.e., only 32, lie along a slightly irregular diagonal line; the gaps between matching segments, which correspond to segments that do not share significant homologies, are relatively wide. The matching segments are so short that they appear as simple dots at a lower magnification (Figure 3A), but at a higher magnification, they appear to be short segments (Figure 3B).

On the contrary, the BLAST 2-nucleotide search of the human GHR intron sequence against the homologous chicken sequence yielded only one 182 nucleotide-long match (i.e., 0.30 of the chicken sequence).

### 3.4. Human/Zebrafish Alignments

**Figure 4:**
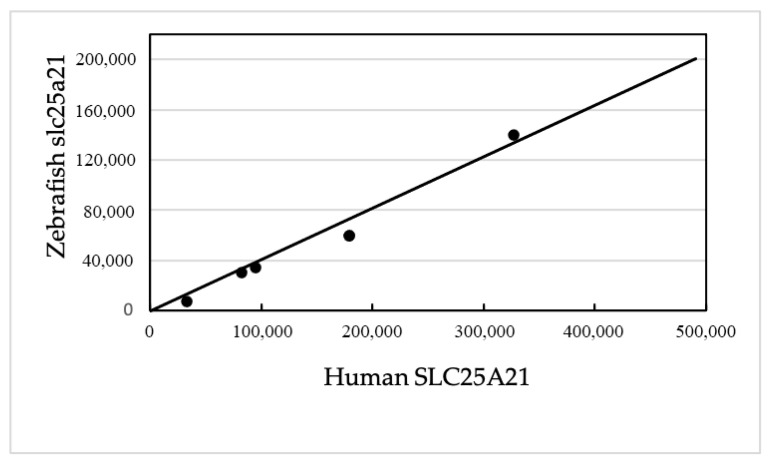
Abscissa: Human SLC25A21 (490,793 nucleotides). Ordinate: Zebrafish slc25a21 (200,517 nucleotides). The matching points significantly align along the diagonal from the lower left corner to the upper right corner. Aligned nucleotides = 1022 over 200,517, i.e., 0.51%.

The BLAST dot plot alignment of the human SLC25A21 with the homologous zebrafish gene yielded five non-TE matches (with a total of 1022 nucleotides; 0.51% of the total) positioned near the theoretical diagonal line (Figure 4).

On the contrary, no significant similarity below the Expect threshold was found when aligning the human GHR intron sequence against the intron sequence of the GHR of zebrafish.

### 3.5. Human/Xenopus Alignments

**Figure 5:**
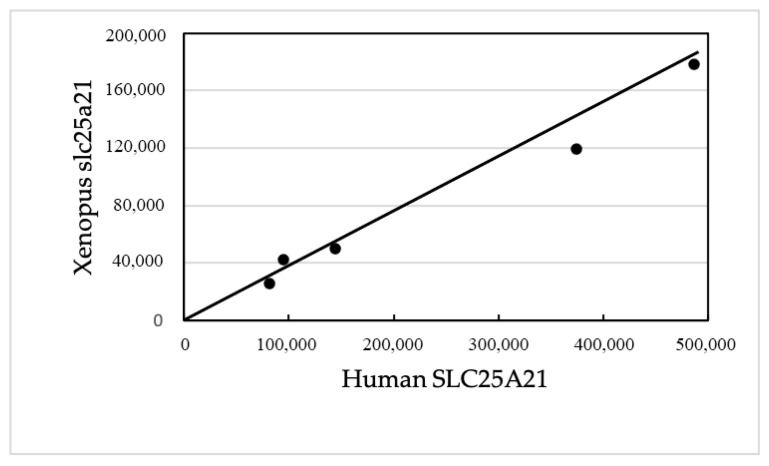
Abscissa: Human SLC25A21 (490,793 nucleotides). Ordinate: Xenopus slc25a21 (187,108 nucleotides). The matching points do not correspond to TEs and significantly align along the diagonal from the lower left corner to the upper right corner. Aligned nucleotides = 846 over 187,108, i.e., 0.45%.

The BLAST dot plot alignment of the human SLC25A21 with the homologous Xenopus gene yielded five non-TE matches (with a total of 846 nucleotides; 0.45% of the total) positioned near the theoretical diagonal line (Figure 5).

On the contrary, the alignment of the human GHR intron sequence against the intron sequence of the GHR of Xenopus did not yield significant similarities below the Expect threshold.

### 3.6. Zebrafish/Chicken Alignments

The BLAST dot plot alignment of the zebrafish slc25a21 with the homologous chicken gene (not shown) yielded five non-TE matches positioned near the theoretical diagonal line. The aligned nucleotides constitute 0.42% of the chicken sequence.

The alignment of the zebrafish GHR intron sequence against the intron sequence of the GHR of chicken did not yield significant similarities.

### 3.7. Zebrafish/Xenopus Alignments

The BLAST dot plot alignment of the zebrafish slc25a21 with the homologous Xenopus gene (not shown) yielded one match only corresponding to 0.11% of the zebrafish sequence and 0.10% of the Xenopus sequence.

The alignment of the zebrafish GHR intron sequence against the intron sequence of the GHR of Xenopus did not yield significant similarities.

### 3.8. Chicken/Xenopus Alignments

The BLAST dot plot alignment of the chicken slc25a21 with the homologous Xenopus gene (not shown) yielded six non-TE matches positioned near the theoretical diagonal line. The aligned nucleotides constitute 0.44% of the chicken sequence.

The alignment of the chicken GHR intron sequence against the intron sequence of the GHR of Xenopus did not yield significant similarities.

### 3.9. Global Analysis of the SLC25A21 and GHR Conservation in Vertebrates

**Figure 6:**
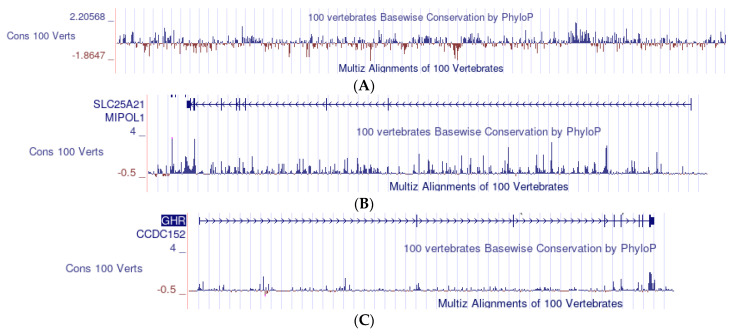
(**A**) An example of the analysis of conservation with the UCSC Genome Browser. The data, plotted as a bar graph, derive from the multialignment of 100 representative vertebrates. Each bar measures the average conservation/mutation of a series of 800 nucleotides; the blue bars (positive values) measure the conservation; the red bars (negative values) measure the mutation; the zero level is the conservation expected under neutral drift on the left of the scale (from −1.8647 to 2.20568). (**B**) SLC25A21: bar graph of the conservation profile in vertebrates. Each of the (minute) bars corresponds to 800 nucleotides. The series of arrows (top of the figure) indicate the direction of translation. The small vertical lines on the line of arrows mark the position of the exons. Other captions are the same as those in (**A**). (**C**) GHR: bar graph of the conservation profile in vertebrates. The same scale and other captions are used in (**B**), but each bar corresponds to 480 nucleotides.

The UCSC Genome Browser, using the function Comparative Genomics/Conservation, plots the estimated evolutionary trend of a given vertebrate gene, calculated along its entire length as an average from 100 multialigned vertebrate species. The system calculates, for each position in the gene, the conservation expected under neutral drift (the zero level in the plot) and then determines the evolutionary “acceleration” (faster evolution than expected, i.e., poor conservation) and the “conservation” (slower than expected evolution) values. The values of “conservation” are plotted as positives, while the values of “acceleration” are plotted as negatives. An example is given in Figure 6A. In addition, the system provides the statistical distribution of the readings and the general average in the section studied.

The conservation profiles of SLC25A21 and GHR are shown in Figure 6B,C, respectively. In both graphs, the positive values largely prevail, although a few small negative peaks may be appreciated in spite of the small enlargement. The general averages of all readings are 0.256 in SLC25A21 and 0.093 in GHR. These averages forcefully include exons; however, these represent a very small fraction of the sequences (0.18% of SLC25A21 in human and 0.66% in GHR). The average figures indicate that, in both genes, the intron sequences tend to be conserved, but the level of conservation is about 2.8 times higher in SLC25A21.

### 3.10. Differences among Species

**Figure 7:**
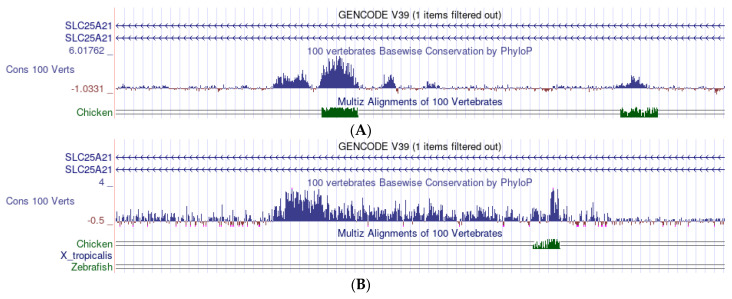
(**A**) A section of the SLC25A21 sequence: The general vertebrate conservation exhibits five distinct peaks, but the chicken sequence matches only two of them. This section approximately corresponds to nucleotides 36,731 K to 36,784 K *in human* (refers to genomic sequences; Table 2). (**B**) A section of the SLC25A21 sequence: the general vertebrate conservation exhibits a long series of conserved nucleotides, while the chicken matches only a small part of the series and zebrafish exhibits no signal at all. This section approximately corresponds *in human* to nucleotides 36,792 K to 36,845 K.

The peaks of conservation in the different vertebrate species do not always match the general vertebrate pattern. Here, we provide two examples covering two different 53,000-nucleotide segments in intron 1 (Figure 7A,B).

### 3.11. Repeats in Introns of Human SLC25A21 and GHR

Within the intronic sequence of human SLC25A21, we found two short Plus/Plus repeats of non-TE sequences: the longest comprised 96 nt (Expect = 5 × 10^−34^). Within the intronic sequence of human GHR, we found four short Plus/Plus repeats of non-TE sequences: the longest comprised 81 nt (Expect = 3 × 10^−22^).

### 3.12. Exon Nucleotide and Amino Acid Conservation in SLC25A21 and GHR (Zebrafish/Human)

In SLC25A21, the zebrafish/human conservation was 69.6% for exon nucleotides and 81.2% for the amino acids. In GHR, the zebrafish/human conservation was 56.3% for exon nucleotides and 43.5% for the amino acids.

## 4. Discussion

Bony fishes (Euteleostomi) appeared about 430 million years ago (MYA) and evolved into Actinopterygii (including zebrafish) and Sarcopterygii. About 350 MYA the Sarcopterygii evolved into Tetrapoda, of which Amphibia (including Xenopus) and Amniota are specific clades. Amniota evolved, possibly around 320 MYA, into Synapsida (ancestors of Mammals) and Sauropsida (comprising Reptilia and Birds). Eventually, rodents/primates divergence took place around 65–100 MYA, while the species Homo sapiens emerged only 160,000 years ago [27,28,29,30,31,32].

Actinopterygii/Sarcopterygii divergence seems to have entailed a radical re-editing of introns. In SLC25A21 alignments of the actinopterygian zebrafish with human, chicken, and Xenopus (all of the sarcopterygian group), there were five (0.51% of the intron sequence), five (0.42%), and one (0.11%) matches, respectively. The corresponding alignments with the less conserved GHR yielded no matches.

In the sarcopterygian group of Tetrapoda, the subsequent Amphibia/Amniota divergence also entailed a similarly radical re-editing of introns. In SLC25A21 alignments of Xenopus with human and chicken, the matches were 5 (0.45% of the intron sequence), and 6 (0.44%), respectively. The corresponding alignments with the less conserved GHR yielded no match.

In Amniota, the Synapsida/Sauropsida divergence appears to be much more conservative. In the SLC25A21 alignment of human with chicken, the matches were 32 (4.66% of the intron sequence). The corresponding alignment with the less conserved GHR yielded only one match (0.30%).

Eventually, as expected, the more recent rodents/primates divergence was more conservative. The human/mouse alignments yielded 266 matches (40.03%) with SLC25A21 and 27 matches (11.07%) with GHR.

The evolution of exons was also more conservative in SLC25A21 than in GHR, as reported in paragraph 3.12. Thus, the nucleotide dynamics of introns seems to parallel that of exons, although changes in introns are possibly less restricted and can accumulate, while exons are subject to a selective pressure, which minimizes changes that would alter their protein product.

A comparison of the total number of intronic nucleotides in human and zebrafish shows that the figures in human are almost three times those in zebrafish, both in SLC25A21 and GHR (Table 3). According to our investigations (unpublished results), through the whole mitochondrial carrier subfamily, the number of intron nucleotides is significantly higher in human than in zebrafish. The reason for a trend of intron length increase during evolution has not yet been completely elucidated. The hypothesis is that introns can grow by progressive addition of new “structure units” at their 3′ end [33]. In addition, the spontaneous duplication of DNA segments, apparently by accidental replication, was described [34]. In the SLC25A3 gene of vertebrates, a whole exon (about 120 nt long) is duplicated, and two copies which retain a high level of similarity are alternatively transcribed, giving rise to two similar transcripts [35,36,37]. In the present material, we found a few short but significant repeats in the human sequences (Section 3.11) that could be the recognizable remnants of larger duplications which had diverged by a summation of mutations. The relatively regular distribution of matches along the entire gene length seems to contradict the hypothesis of additions at the 3′ end only; on the other hand, the number of duplications observed appears too low to support weighty intron elongations. These observations suggest that the increase in intron length during evolution may occur through different mechanisms.

## 5. Conclusions

Using the NIH Nucleotide BLAST suite to align the introns of homologous genes of two species, the “dot plot” provides a complete visual image of the intron conservation between the two species. Aligning a genuine intronic sequence with the same sequence after TEs clearing, the “dot plot” returns a clear (negative) image of the numerousness and positions of TEs (Figure 1E). However, when the two species share similar TEs, as is the case of the human SINE *Alu* and mouse SINE *B1* [38], several matches show outside of the alignment diagonal (Figure 2A), but after TE removal, these matches are no longer present (Figure 2B). The UCSC Genome graphics suite may be a powerful tool for displaying multicomparisons along the entire gene of reference, the average conservation level in vertebrates and the conservation level profile in selected individual species, simultaneously. Thus, the combination of the two approaches may be helpful to gain a better understanding of the general evolutionary dynamics in vertebrate introns. For this connection, the graphically basic approaches outlined here may help in providing a qualitative, but readily visual, evaluation of the intron structural conservation/mutation and integration with transposable elements in vertebrate evolution, as a preliminary step to more quantitative approaches.

## Figures and Tables

**Table 1:** The transcripts and their mRNAs (NCBI GenBank).

		Transcript	Exons	Sequence ID	mRNA
				mRNA	Nucleotides
SLC25A21	Human	Variant 1	10	NM_030631.4	900
SLC25A21	Mouse	Variant 2	10	NM_001167976.1	918
SLC25A21	Chicken	Variant X2	11	XM_025150918.1	1002
SLC25A21	Zebrafish	-----------	10	NM_001076632.1	897
SLC25A21	Xenopus	-----------	10	NM_001017069.2	900
GHR	Human	Variant 2	9	NC_000005.10	1938
GHR	Mouse	Variant 4	10	NM_001286370.1	1953
GHR	Chicken	-----------	8	NM_001001293.1	1827
GHR	Zebrafish	-----------	8	NM_001083578.1	1713
GHR	Xenopus	-----------	9	XM_002933047.4	1866

**Table 2:** The genomic sequences (NCBI Reference Sequence). Coverage: FROM the first nucleotide of the Start codon to the last nucleotide of the Stop codon.

		Chromosome	Sequence ID	FROM	TO
			Genomic		
SLC25A21	Human	14	NC_000014.9	36,680,658	37,172,350
SLC25A21	Mouse	12	NC_000078.7	56,760,598	57,206,029
SLC25A21	Chicken	5	NC_006092.5	36,947,211	37,178,401
SLC25A21	Zebrafish	17	NC_007128.7	38,030,627	38,232,040
SLC25A21	Xenopus	8	NC_030684.2	108,640,883	108,828,890
GHR	Human	5	NC_000005.10	42,424,589	42,719,424
GHR	Mouse	15	NC_000081.7	3,349,224	3,487,481
GHR	Chicken	Z	NC_006127.5	13,495,919	13,558,411
GHR	Zebrafish	8	NC_007119.7	31,465,534	31,571,560
GHR	Xenopus	1	NC_030677.2	195,152,927	195,320,257

**Table 3:** Total: total number of nucleotides (start to stop codons); Intronic: number of intronic nucleotides; Transposable Elements: % of nucleotides of transposable elements over the total intronic nucleotides; Intronic Non-TE: intronic nucleotides minus TE nucleotides.

		Total	Intronic	Transposable	Intronic
		nt	nt	Elements %	Non-TE nt
SLC25A21	Human	491,693	490,793	39.24	298,209
SLC25A21	Mouse	445,432	444,514	33.33	296,350
SLC25A21	Chicken	231,191	230,189	12.97	200,341
SLC25A21	Zebrafish	201,414	200,517	49.02	102,233
SLC25A21	Xenopus	188,008	187,108	41.23	109,972
GHR	Human	294,836	292,898	44.09	163,758
GHR	Mouse	138,258	136,305	34.14	89,777
GHR	Chicken	62,493	60,666	15.05	51,537
GHR	Zebrafish	106,027	104,314	74.84	26,244
GHR	Xenopus	167,331	165,465	43.06	94,223
			2,312,769		1,432,644

## Data Availability

Data and methods used in this paper are presented in sufficient details. Any additional questions should be directed to the authors.
